# A Single Session of Whole-Body Electromyostimulation Increases Muscle Strength, Endurance and proNGF in Early Parkinson Patients

**DOI:** 10.3390/ijerph18105499

**Published:** 2021-05-20

**Authors:** Giovanni Fiorilli, Federico Quinzi, Andrea Buonsenso, Giusy Casazza, Luigi Manni, Attilio Parisi, Alfonso Di Costanzo, Giuseppe Calcagno, Marzia Soligo, Alessandra di Cagno

**Affiliations:** 1 Department of Medicine and Health Sciences, University of Molise, 86100 Campobasso, Italy; fiorilli@unimol.it (G.F.); andreabuonsenso@gmail.com (A.B.); giusy.casazza93@gmail.com (G.C.); alfonso.dicostanzo@unimol.it (A.D.C.); 2 Department of Motor, Human and Health Sciences, University of Rome “Foro Italico”, 00197 Rome, Italy; fquinzi@libero.it (F.Q.); attilio.parisi@uniroma4.it (A.P.); alessandra.dicagno@uniroma4.it (A.d.C.); 3 Institute of Translational Pharmacology, National Research Council, 00133 Rome, Italy; luigi.manni@ift.cnr.it (L.M.); marzia.soligo@ift.cnr.it (M.S.)

**Keywords:** Parkinson’s disease, motor impairment, physical activity, neurotrophic factors

## Abstract

Parkinson’s disease (PD) patients lead a sedentary lifestyle, being unable or unwilling to exercise conventionally, due to physical and mental limitations. The aim of this study was to assess the acute effects of a single session of whole-body electromyostimulation (WB-EMS) on the physical performances and serum levels of the neurotrophic factors in PD patients. Ten subjects (aged 72.60 ± 6.82) underwent 20 min of physical activity with superimposed WB-EMS and, after four weeks, the same protocol with no WB-EMS. WB-EMS was conducted with intermittent stimulation, with 4 s WB-EMS/4 s rest, at 85 Hz, 350 μs. A physical fitness assessment and blood samples collection, to evaluate neurotrophic factors’ levels (BDNF, FGF21, proNGF, mNGF), were collected before and after the intervention. The RM-ANOVA showed significant improvements in sit-to-stand (*p* < 0.01), arm curl (*p* < 0.01), handgrip (*p* < 0.01) and soda pop test (*p* < 0.01) after the WB-EMS intervention. Higher proNFG serum levels were observed in the WB-EMS condition compared to the no WB-EMS after 60 min post-intervention (*p* = 0.0163). The effect of WB-EMS confirmed the electrostimulation ability to modulate the proNGF quantity. The positive impact of the WB-EMS protocol on physical functioning, and eye–hand coordination, makes this intervention a promising strategy to improve motor and non-motor symptoms in PD patients.

## 1. Introduction

Parkinson’s disease (PD) is the fastest growing disability, which leads to hospitalization and death [1]. PD is a neurodegenerative chronic disease characterized by motor and non-motor impairments [2]. The main visible motor impairments include difficulties in initiating movements (akinesia) and slowness and difficulty in maintaining movements (bradykinesia), postural and gait instabilities, and muscle rigidity and tremors [3]. Moreover, non-motor symptoms, such as pain, cognitive impairment and dementia, apathy, and general fatigue [4,5], affect most of the patients. The onset of the disease is mainly due to the rupture of nigro-striatum-thalamus-cortical circuits and catecholaminergic deficit, in particular of the neurotransmitter dopamine [6].

Lifestyle factors influence the development of PD [7], for example, nursing home placement leads to cognitive impairment and non-dopaminergic motor deficit [8]. Conversely, physical activity (PA) could prevent the loss of dopaminergic neurons and improve synaptic connections, upregulating neurotrophic factor levels and consequently counteracting dyskinesia [9,10]. 

The exercise program usually proposed to Parkinson’s patients includes moderate intensity aerobic activity to maintain the patients’ cardiorespiratory fitness and brain resting functional efficiency, such as functional connectivity between the frontoparietal network and the fronto-executive network [11,12]. 

However, resistance training of large muscle groups improves motor symptoms such as the rate of force development, balance, reaction time, and gait speed [13]. Previous studies highlighted that resistance training improves also non-motor symptoms such as attention and working memory [14], minimizing the secondary complications of Parkinson’s disease such as hyperexcitability of striatal neurons after dopamine depletion [15], osteoporosis, and sarcopenia. This kind of exercise promotes wellbeing [16] and turns out to be more suitable for Parkinson’s patients [17,18] than other exercise modalities. Despite all these, Parkinson’s patients tend to lead a sedentary lifestyle, due both to physical and mental limitations [19]. Parkinson’s patients may be scarcely motivated to engage in healthy habits, including PA, as it presents difficulties in changing their sedentary behavior. Therefore, motivation would be an important factor to be stimulated in PA engagement, promoting adherence and a more physically active lifestyle [20]. Early intervention is crucial in maintaining physical performance and PA programs have to be continuous to guarantee exercise benefits [21]. 

Nevertheless, it is mandatory to identify which kind of PA could be safer and more suitable for PD patients. Mc Gill [22] recently proposed dance as an alternative form of PA for PD patients, improving balance, gait variables, and depression and anxiety, common in PD. Schenkman [23] showed promising results with high-intensity exercise on the treadmill. The use of technology to enhance and potentiate the PA effect is largely spread both in prevention and rehabilitation programs [24].

Whole-body electromyostimulation (WB-EMS) seems a promising exercise modality for people unable or scarcely motivated to exercise conventionally [25]. WB-EMS induces a global-body electrical myostimulation, activating up to 8–10 different muscle groups synchronously, and it allows exercising a given kinetic chain while simultaneously performing functional movements during the stimulation. Last, WB-EMS is a time-saving training methodology, especially for people who did not have the ability to perform prolonged physical exercise [26]. In fact, this kind of regimen could be both time-saving and less debilitating than traditional PA, and consequently well tolerated, producing adherence among sedentary subjects. It is well known that PA has to be continuous to guarantee its effectiveness. The subjects undergoing this type of intervention could show great engagement, perceiving fast improvements [27] leading to an increase in self-efficacy. As a training tool, WB-EMS is orthopedically gentle and offers good results on body composition, strength improvements by neuromuscular adaptations, intermuscular coordination and muscle size, and increases energy consumption [28]. These improvements could be optimal in preserving independence. Furthermore, exercise superimposed WB-EMS has been shown to modulate serum proteins, such as the neurotrophin brain-derived neurotrophic factor (BDNF) [29], which have been indicated as potential biomarkers related to disease progression and the severity of motor impairment in PD patients [30]. No previous studies on neurological disease patients applied WB-EMS, which may be functional in counteracting the physical inactivity secondary to the associated disability [19].

Therefore, the primary aim of this study was to assess the acute effects of a bout of WB-EMS exercise on the physical performances and serum levels of neurotrophic factors in Parkinson’s patients. The second aim was to assess if the protocol of WB-EMS combined with voluntary strength exercises, proposed in this study, could be safe and suitable for Parkinson’s patients, considering their characteristics and their degree of satisfaction, which will guarantee their adherence. 

WB-EMS has not previously been applied to an exercise program for PD patients. The initiative to develop WB-EMS training protocols was motivated by the awareness that Parkinson’s patients are unable or unwilling to perform traditional exercise programs, and WB-EMS training could be a compensatory non-pharmacologic strategy to delay the progression of the decline in physical and cognitive functions, promoting an acceptable quality of life [31].

## 2. Materials and Methods

### 2.1. Study Design

The present study was designed as a randomized controlled trial in which subjects received two treatments, aimed to evaluate the acute effects of WB-EMS intervention, compared with the same protocol with no WB-EMS, on physical performances and neurotrophic factors levels in subjects with PD.

### 2.2. Participants

Twelve subjects (aged 72.60 ± 6.82) were recruited for the present study from community centers for elderly of Campobasso. Two subjects dropped out before the second phase of the intervention. The sample characteristics are showed in Table 1. 

To be eligible for the study, participants were required to meet the following inclusion criteria: (a) age from 50 to 80 years; (b) clinical diagnosis of PD in the stage from 1 (mild) to 3 (moderate) assessed by the Hoehn and Yahr scale [32,33]; (c) no physical exercise program attendance. Exclusion criteria included the following: (a) mini-mental state examination (MMSE) score of less than 24 [34]; (b) inability to walk for 6 min without assistance [35]; (c) the presence of a medical condition influencing the cognitive and/or motor functions; (d) presence of any counterindication for the utilization of EMS. Participants maintained their pharmacological (levodopa) treatment. 

After a careful explanation of the aims, the risks and the benefits of the study, all participants signed the written informed consent. The study was designed and conducted in accordance with the Declaration of Helsinki and approved by the Bioethical Committee “Azienda Sanitaria Regionale Molise—ASREM” (11487/2020).

### 2.3. Experimental Procedures

Participants performed WB-EMS intervention first and, after four weeks, the protocol was repeated with no WB-EMS, as the control condition. All participants attended the interventions at the same time of the day. A four-week wash-out period was planned between the two interventions to reduce possible carry-over effects.

**Figure 1:**
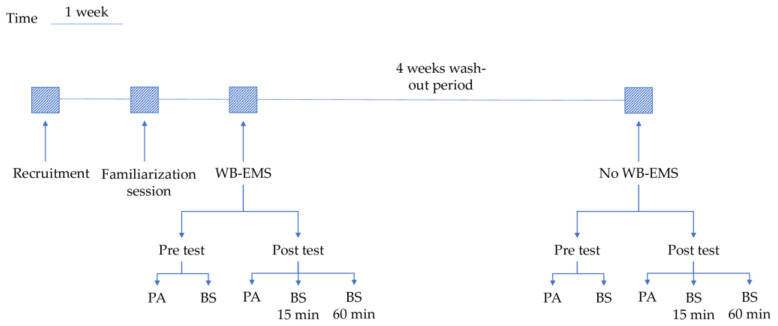
Diagram of experimental procedure. PA: physical assessment; BS: blood samples collection.

A familiarization session was performed one week before the first intervention. This session aimed to learn movement patterns (i.e., proper techniques of the exercises) and to adapt participants to the electric stimuli. Each muscle group was strained in intervals to determine each participant’s subjective maximum. The maximum was determined by the point at which each person gave the signal to stop the strain, such as the highest degree of strain that may be easily tolerated. The last stimulation value output by the EMS was recorded [36]. The diagram of the experimental procedure is showed in Figure 1.

**Figure 2:**
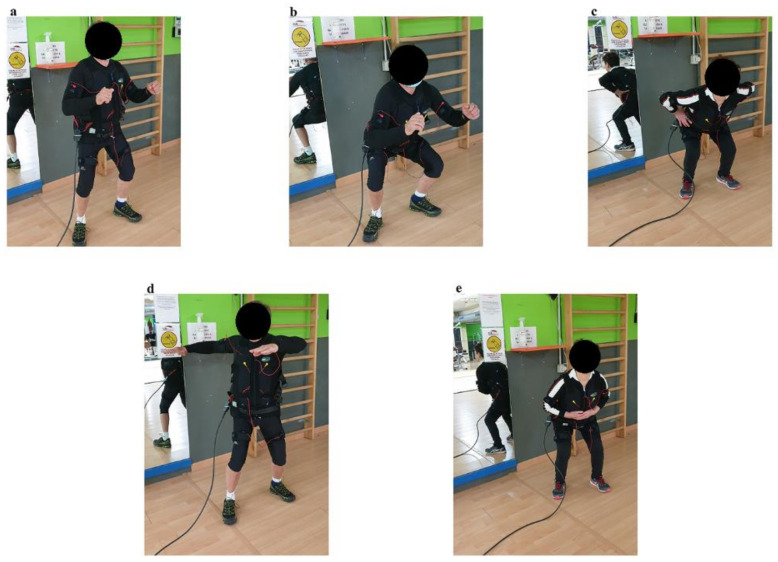
Workout session. (**a**) Half squat; (**b**) full squat; (**c**) bent over; (**d**) core rotation; (**e**) crunch.

In both WB-EMS and no WB-EMS interventions, participants underwent a 20-min workout session guided and supervised by a certified instructor. The workout session consisted of the following 5 voluntary strength exercises: half squat, full squat, bent over, core rotation and crunch (Figure 2). After 5 min of warm up, each exercise was performed for 3 min, during which participants were required to perform alternatively 4 s of isometric contraction and 4 s of static rest.

In the WB-EMS intervention, the 20-min workout session combined the 5 voluntary strength exercises to a superimposed and supervised WB-EMS application, using the protocol (rectangular stimulation at 75–85 Hz, 350 μs, 4 s stimulation/4 s rest) recommended by Kemmler et al. [37]. Participants were encouraged to perform with maximal effort during the WB-EMS impulse. In order to generate a sufficient but tolerable intensity, the WB-EMS impulse was monitored according to the Borg’s rating of perceived exertion (RPE), a 6–20 points scale [38], on which a score <11 is considered an effort equal to a low intensity exercise, 12–13 to a moderate exercise, and 14–17 to a vigorous exercise [39]. Current intensity was individually adapted for each region during the familiarization session to an RPE of 13–16 of 20 (from moderate to vigorous).

### 2.4. Experimental Set Up

The WB-EMS application was conducted with Miha Bodytec equipment (GmbH, Augsburg, Germany). Miha Bodytec consists of a central unit that activates 10 channels with a rectangular two-phase asymmetrical current stimulation, and treatment vest where the electrodes are placed (bio-jacket). This equipment simultaneously activated 10 major muscle groups, including muscles of upper arms, chest, shoulders, upper and lower back, abdominals, gluteal, hip region including the upper limbs. Miha Bodytec EMS provides complete freedom of movement for the participants. 

### 2.5. Baseline Assessment

For the baseline assessment, the following information was collected: socio-demographic data such as age and gender, the physical activity scale for the elderly (PASE), and the unified Parkinson’s disease rating scale (UPDRS).

Successively cognitive performances, physical fitness level, and neurotrophic factors levels were assessed pre- and post-intervention. After the familiarization session at baseline, participants underwent 2 testing sessions. Before the intervention, physical tests were administered and blood samples were collected before the protocol, after 15 min and after 60 min. 

#### 2.5.1. Physical Fitness Assessment

The general level of PA was assessed with the PASE [40]. This is a questionnaire consisting of 12 items self-administered, evaluating PA performed over the previous week during leisure time, household and work activities. A score, ranging from 0 to 793, with higher scores indicating greater PA was assigned [41]. The obtained participants score was 69.31 ± 47.86, meaning low level of PA. 

To assess the physical functions, the following tests were administered: Arm tremor to evaluate the degree of Parkinson’s-related tremor, assessed via app (Accelerometer Data Recorder) on the arm with higher level of tremor. Upper limb endurance was evaluated by asking the participants to perform the maximal number of repetitions of the arm curl exercise with a weight of 3 kg for men and 2 kg for women [42]. Upper limb strength was evaluated using the handgrip strength test [43]. The arm curl and the handgrip tests were bilaterally performed. 

Moreover, participants underwent the following tests: sit-to-stand test for lower limbs’ endurance [44], 8 feet up and go for dynamic balance and agility skills [45], six minutes walking test to assess the cardiorespiratory fitness [35], chair sit and reach test for trunk and lower limbs’ flexibility [46], soda pop test for the oculo-manual coordination [47], Tinetti balance and gait test for balance, walking and fall-risk evaluation [48]. 

#### 2.5.2. Neurotrophic Factors Serum Levels

Brain-derived neurotrophic factor (BDNF) or nerve growth factor (NGF) have been indicated as potential serum biomarkers that correlate with disease severity, motor and cognitive performance in PD patients [49]. For other growth factors, such as fibroblast growth factor-21 (FGF-21), despite being identified as potentially relevant serum biomarkers, controversial data has been produced so far [50]. In the present work, ELISAs were used to evaluate possible serum variations in these growth factors after acute WB-EMS. Blood samples were collected from the antecubital vein before training, and 15 and 60 min after training. The blood was centrifuged (Eppendorf Centrifuge 5804) for 15 min at 1200 relative centrifugal force. The supernatant was decanted and stored in a −80 °C freezer until analysis. The sample was analyzed at the Institute of Translational Pharmacology, National Research Council (IFT-CNR) in Rome. Total BDNF content was measured by Duoset^®^ human BDNF ELISA (cat. DY248 R&D Systems), according to manufacturer’s instructions. Total FGF-21 content was measured by Duoset^®^ human FGF-21 ELISA (cat. DY2539 R&D Systems), according to manufacturer’s instructions. Total proNGF and mNGF content were measured according to Soligo et al. [51].

### 2.6. Statistical Analysis

All the statistical analyses detailed in the following paragraph have been carried out using the Statistica software (v.10, StatSoft Inc. Tulsa, OK, USA). All variables have been tested for normal distribution using the Shapiro–Wilk test. For all statistical tests α level has been set to 0.05.

Physical variables (tremor, sit-to-stand test, soda pop, 8 feet up and go, six minutes walking test, chair sit and reach test, Tinetti balance and gait test) showed a normal distribution and were submitted to separate RM-ANOVA with intervention (WB-EMS vs no WB-EMS) and time (pre- vs. post-intervention) as repeated factors. Arm curl and handgrip tests were submitted to separate RM-ANOVA with intervention, time and side (right vs. left). Neurotrophic factors (BDNF, FGF21, proNGF, mNGF) were submitted to separate RM-ANOVA with intervention and time as repeated factors. Neurotrophic factors were analyzed also in terms of increments both in absolute (^Δ^BDNF, ^Δ^FGF21, ^Δ^proNGF, ^Δ^mNGF) and in percentage of the initial assessment (^%^BDNF, ^%^FGF21, ^%^proNGF, ^%^mNGF). Tukey’s honestly significant difference post-hoc test was used where appropriate.

Scales for the assessment of the disease (UPRDS and PASE) did not show a normal distribution and the effects of intervention were verified by means of a non-parametric test (the Wilcoxon matched pair test).

## 3. Results

### 3.1. Physical Assessment

The RM-ANOVA performed on tremor, 8 feet up and go, the six minutes walking test and Tinetti balance and gait test showed no significant effect of intervention, time or interaction effects (all *p*s > 0.05). A significant effect of time was observed for the chair sit and reach test. The Tukey’s post-hoc test showed that the participants in the pre-intervention assessment had higher flexibility than in the post-intervention assessment (*p* = 0.018). 

The RM-ANOVA showed a significant intervention by time interaction for the sit-to-stand test. The post-hoc test showed that in the post-WB-EMS condition, the participants were able to perform more repetitions than in the post-no WB-EMS condition (*p* = 0.0008) and in the pre-WB-EMS (*p* < 0.001) condition.

Similarly, a significant intervention by time interaction was observed for the soda pop test. The post-hoc showed that in the post-WB-EMS condition, the participants were faster than in the post-no WB-EMS condition (*p* = 0.0068). In addition, the participants were slower in the post-no WB-EMS condition than in the pre-WB-EMS condition (*p* = 0.033).

The RM-ANOVA performed on the arm curl test showed a significant intervention by time interaction and the post-hoc test revealed that in the post-WB-EMS condition, the participants were able to perform a larger number of repetitions than in the post-no WB-EMS (*p* = 0.008) and in the pre-WB-EMS (*p* = 0.0007). No significant effect of the side of body was observed for arm curl (F = 2.42; *p* = 0.15). 

**Figure 3:**
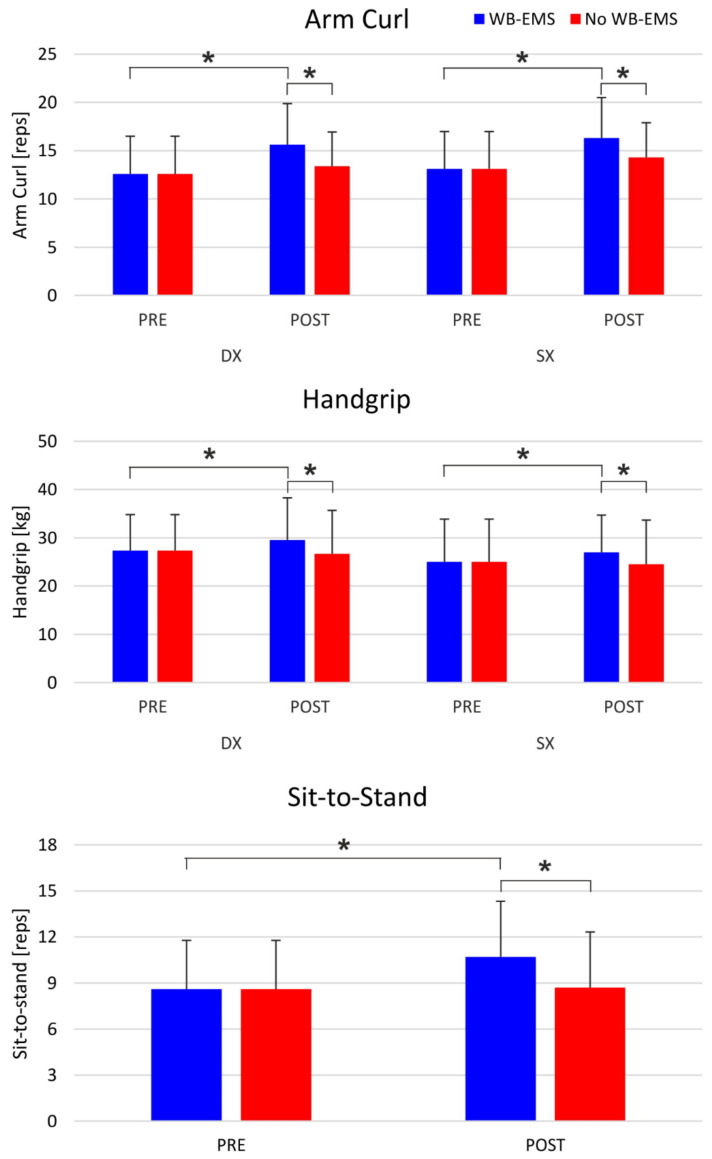
Physical performances: comparison between WB-EMS intervention and no WB-EMS intervention and pre- and post-assessment. * *p* < 0.05; reps: repetitions; kg: kilograms.

Also, for the handgrip test the RM-ANOVA showed a significant intervention by time interaction. The Tukey’s post-hoc showed that after the WB-EMS intervention (post-WB-EMS), the participants were stronger than after the control condition (post-no WB-EMS; *p* = 0.009) and before the WB-EMS treatment (pre-WB-EMS; *p* = 0.036). A significant effect of the side was observed for this variable, with the right arm being stronger than the left one (*p* = 0.048) (Figure 3). The results of the physical assessment are reported in Table 2.

### 3.2. Neurotrophic Factors

**Figure 4:**
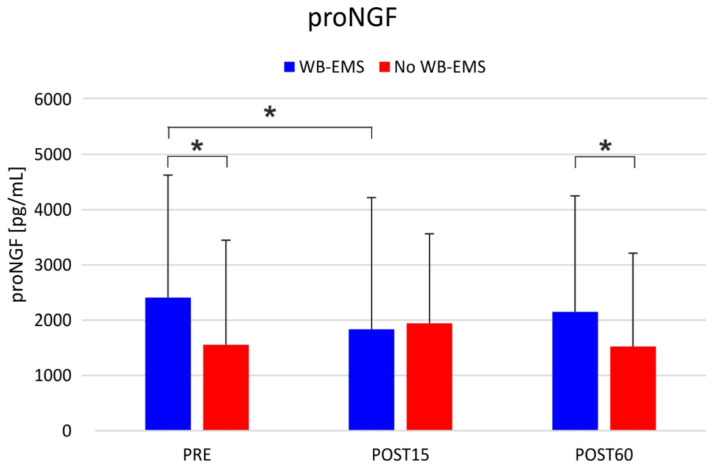
Level of proNGF variations: comparison between WB-EMS intervention and no WB-EMS intervention and pre- and post-assessment (15 and 60 min). * *p* < 0.05; pg/mL: picograms/millimeters.

The RM-ANOVA did not show any significant effect of intervention and time for BDNF, FGF21, and mNFG. Conversely, a significant intervention by time interaction was observed for proNFG, with higher serum levels in the WB-EMS than in the no-WB-EMS at the pre-intervention assessment (*p* = 0.0011 in the post-hoc comparison) and 60 min post-intervention (*p* = 0.0163). In the WB-EMS condition, lower levels of proNGF were observed 15 min after the WB-EMS intervention compared to the baseline values (*p* = 0.0315) (Figure 4).

When the variations in the neurotrophic factors were expressed as a percentage of the pre-intervention value (pre-intervention assessment), there was no effect of intervention or time and no significant interactions emerged for ^%^BDNF, ^%^FGF21, ^%^proNGF, and ^%^mNGF. Conversely, when absolute pre- and post-interventions were considered, a significant intervention by time interaction emerged for ^Δ^proNGF with decrements (−574.6 pg/mL) observed 15 min after the WB-EMS treatment, whereas in the no-WB-EMS after 15 min an increment was observed (390.2 pg/mL; *p* < 0.001). Sixty minutes after the treatment, WB-EMS and no WB-EMS showed comparable variations (WB-EMS: −256.9 pg/mL vs. no WB-EMS: −30.0 pg/mL). In addition, a significant effect of intervention was observed for ^Δ^proNGF, with WB-EMS showing a decrement (WB-EMS: −415.9 pg/mL) of this neurotrophic factor compared to the no WB-EMS condition where an increase could be observed (CON: 180.1 pg/mL; *p* = 0.028). The analysis of the increments showed no significant main effects or interactions. The results of the neurotrophic factors are reported in Table 3 and Table 4.

### 3.3. Scales for the Assessment of the Pathology

The Wilcoxon matched pair test showed no difference between the WB-EMS and no WB-EMS conditions in UPDRS total (*p* = 0.123).

## 4. Discussion

This is the first study in which WB-EMS was applied to neurodegenerative patients such as PD patients. 

After a single session of WB-EMS, significant improvements in upper and lower limbs strength (arm curl and handgrip, sit-to-stand) were observed, while no significant results were observed for the 8 feet up and go test. Probably, for the latter, the discontinuous pattern of this exercise (standing, walking, turning, sitting) might have accounted for the non-significant results as it represents a great challenge related to the PD disease at the basal ganglia level [9]. It was expected that an increase in muscle endurance and strength could be very promising, especially when considered in the framework of chronic training. Indeed, muscle strengthening, by promoting muscle hypertrophy, counteracts the loss of muscle mass, commonly observed in the elderly, which leads to a worsening in gate, mobility and posture [52].

No significant results were found in the six minutes walking test and Tinetti balance and gait test, after a single session both with and without the WB-EMS application. Walking is particularly effected in PD and its decline precedes other gait-dependent limitations [53]. A previous study showed that psychological factors, such as low self-efficacy and low expectation of success, were the primary factors limiting walking more than motor impairments [54]. In addition, it cannot be excluded that fatigue might have compromised the result of these tests. Fatigue is the most common non-motor symptom, strongly experienced by 33–58% of PD patients [55,56], and previous studies showed that changes in PA levels did not reduce the perception of fatigue in PD patients [57,58]. Moreover, fear of falls might increase the gait and walking difficulties [59]. From the above mentioned considerations, we argued that extensive core and lower limb-oriented strength training, performed before any type of exercise, could facilitate better performances [60] and changes in self-confidence [61]. 

Considering that, in the early stages of PD, abnormalities linked to bradykinesia [62] occurred in hand–eye coordination and in the prolonged reaction time [63], it was expected that the level of oculo-manual coordination would not improve after conventional exercise. However, in the soda pop test, a test used to assess manual dexterity and hand–eye coordination, the significant differences observed between WB-EMS and no WB-EMS conditions were probably due to the neural adaptation observed in other studies after the exposure to WB-EMS [64,65]. Consequently, it can be assumed that WB-EMS may positively affect the participants’ reaction time. Further studies might verify and confirm this hypothesis.

The chair sit and reach assessment showed a worsening in both the two intervention conditions. It could be explained by the enhancement of muscle tone that occurs after a resistance session, or no optimal muscle strain [66]. For further applications it could be advisable to schedule a flexibility session at the end of the WB-EMS resistance training.

The outcome measures related to tremor symptoms did not show significant improvements after the two conditions (WB-EMS and no WB-EMS). A previous review revealed poor PA effects on PD clinical symptoms. It is widely recognized that physical fitness and general health benefit more rapidly and positively from PA intervention than the other PD clinical symptoms [67]. Persistent and long duration PA could ensure greater effects [68]. Moreover, it could be considered that PD symptoms are variable over time, from day to day and even within one day, therefore it is possible that the pre- and post-assessments occurred in brief moments and were not be able to capture positive variation in those small measurement times [69]. 

In this study, we found that a single session of WB-EMS is able to significantly modify serum proNGF levels only. The lack of effects recorded for BDNF, a serum biomarker in PD [70] also known to be modulated by physical therapies and electrostimulation [71], may depend on whether the therapeutic treatment was given in a single administration. Furthermore, the relatively low number of patients enrolled constituted a limitation of the present study, and this is reflected in the high standard deviation values obtained and reported in Table 3 and Table 4. Further studies are ongoing to evaluate the effects of prolonged exposure to WB-EMS on serum levels of BDNF in a PD patient larger cohort. Moreover, our data seem to confirm that FGF-21 does not constitute an effective biomarker in PD patients [50] and that a single WB-EMS does not modulate its serum levels. 

On the other hand, our data seem to confirm the recently reported finding about the value of proNGF as an effective serum biomarker in PD [72]. Regarding the data presented for proNGF, the superimposition of WB-EMS on physical exercise changed the sign of the variations recorded in the short-time interval of 60 min considered. This indicates a specific effect of the myostimulation intervention, an effect predicted on the basis of previous observations about the ability of muscle electrostimulation to modulate the tissue quantity of proNGF [73]. In addition, the proNGF values in the serum of the control patients and those treated with WB-EMS before the therapies (pre) differ from each other, the latter being significantly higher than the former. This seems to be an apparent inconsistency, given that the one-month wash-out period between the control session and the WB-EMS session is sufficient to eliminate any effects of mere physical exercise. This inconsistency may be explained in two ways. First, an effect of the patients’ expectation regarding WB-EMS. It has already been reported that serum NGF levels can be increased by the expectation of a physical challenge or a stressful event, correlating with the increase in blood cortisol [74]. Second, the familiarization session with the WB-EMS therapy, carried out by applying electric stimulations a few days before the WB-EMS therapy session, had the effect of increasing the basal serum proNGF levels. This hypothesis is supported by previous studies demonstrating the efficacy of low-frequency electrostimulation in modulating both gene expression and NGF protein content in peripheral tissues [75].

### Limitations

The findings of this study should be viewed in the context of the following limitations:

- Small number of enrolled participants, with different characteristics and PD symptoms;
- Data collected are not generalizable to all clinical diagnosis of PD patients since participants included in this study were in the stage from mild (1) to moderate (3) assessed by the Hoehn and Yahr scale;
- The acute effects of physical activity with superimposed WB-EMS were evaluated. Further studies will assess long-term effects of the WB-EMS protocol on PD patients;
- Further studies will confirm these outcomes by enrolling a higher number of PD patients, in order to obtain a more reliable statistical result.

## 5. Conclusions

The results revealed a positive impact of the WB-EMS protocol proposed in this study, on several physical functioning parameters, improving upper and lower limb strength, hand–eye coordination and clinical outcomes associated with WB-EMS-induced variations in serum proNGF levels.

WB-EMS application could be an advisable strategy for Parkinson’s patients to increase their adherence to PA programs, since it is a time-efficient and feasible methodology to improve their physical condition. Based on this preliminary evidence, further studies on this topic will be set up for a longer time (chronic condition) to compare different modalities of physical exercise combined with myoelectric stimulation, which certainly leads to fruitful outcomes, both on clinical outcomes and on serum biomarker levels in PD patients.

## Figures and Tables

**Table 1:** Sample characteristics.

Variables	Mean ± SD
Age	72.60 ± 6.82
Gender (*n*)	
Males	6
Females	4
Hoehn and Yahr scale	1.60 ± 0.52
UPDRS score	40.20 ± 20.67
MMSE score	26.60 ± 1.26
PASE score	69.31 ± 47.86

**Table 2:** Physical assessment variables. Data as mean and SD.

	WB-EMS		No WB-EMS		Intervention	Time	Interaction
	Pre	Post	Pre	Post	F (1,9)	F (1,9)	F (1,9)
Tremor (Hz)	48.8 (0.7)	48.3 (0.6)	48.8 (0.7)	48.4 (0.6)	F < 1; *p* = 0.86	F = 3.3; *p* = 0.10	F < 1; *p* = 0.86
Sit-To-Stand (reps) *^, §, #^	8.6 (3.2)	10.7 (3.6)	8.6 (3.2)	8.7 (3.6)	F = 20.1; *p* < 0.001	F = 12.9; *p* < 0.01	F = 20.0; *p* < 0.01
Soda pop (s) *^, #^	12.2 (5.4)	11.5 (6.5)	12.2 (5.4)	14.4 (8.5)	F = 10.1; *p* = 0.011	F < 1; *p* = 0.35	F = 10.1; *p* = 0.011
8-feet up & go (s)	16.5 (12.0)	14.2 (11.5)	16.5 (12.0)	17.5 (14.3)	F = 1.24; *p* = 0.29	F = 0.15; *p* = 0.69	F = 1.24; *p* = 0.29
6-mwt (m)	308.7 (132.0)	310.2 (131.8)	308.7 (132.0)	295.2 (128.5)	F < 1; *p* = 0.39	F < 1; *p* = 0.66	F < 1; *p* = 0.39
Chair sit and Reach (cm)	−6.1 (8.0)	−3.4 (8.6)	−6.1 (8.0)	−4.9 (8.8)	F < 1; *p* = 0.56	F = 8.3; *p* = 0.0181	F < 1; *p* = 0.56
Tinetti (score)	24.0 (4.2)	25.0 (3.7)	24.0 (4.2)	23.8 (4.6)	F = 3.8; *p* = 0.081	F = 1.0; *p* = 0.34	F =3.8; *p* = 0.081
Arm Curl (reps) *^, §, #^	12.8 (3.8)	15.9 (4.1)	12.8 (3.8)	13.8 (3.5)	F = 9.5; *p* = 0.0127	F = 5.4; *p* = 0.0440	F = 9.5; *p* = 0.013
Handgrip (kg) *^, #^	26.2 (8.0)	28.3 (8.1)	26.2 (8.0)	25.6 (8.8)	F = 9.0; *p* = 0.0145	F = 1.4; *p* = 0.26	F = 9.0; *p* = 0.014

* Denotes significant effect of treatment; ^§^ denotes significant effect of time; ^#^ denotes significant interaction; Hz: Hertz; reps: repetitions; s: seconds; m: metres; cm: centimeters; kg: kilograms.

**Table 3:** Neurotrophic factor variables. Data as mean and SD.

	WB-EMS			no WB-EMS			Intervention	Time	Interaction
	Pre	Post15	Post60	Pre	Post15	Post60	F (1,9)	F (2,18)	F (2,18)
BDNF (pg/mL)	2243.5 (1050.7)	2247.7 (962.0)	2353.8 (1144.0)	2317.7 (980.1)	2448.1 (949.1)	2419.8 (949.1)	F = 1.41; *p* = 0.264	F = 2.47; *p* = 0.112	F = 1.10; *p* = 0.352
FGF21 (pg/mL)	715.6 (477.5)	663.9 (427.0)	688.0 (379.7)	697.6 (383.5)	972.1 (648.9)	776.8 (440.2)	F = 2.27; *p* = 0.165	F = 1.14; *p* = 0.339	F = 3.14; *p* = 0.067
proNFG (pg/mL) *^, #^	2408.2 (2219.2)	1833.5 (2388.0)	2151.2 (2099.9)	1552.6 (1896.2)	1942.8 (1622.6)	1522.6 (1689.8)	F = 4.1; *p* = 0.074	F < 1; *p* = 0.503	F = 8.9; *p* = 0.002
mNFG (pg/mL)	78.0 (485.3)	42.8 (439.2)	33.4 (400.5)	43.2 (426.5)	−18.3 (239.2)	−2.9 (279.2)	F < 1; *p* = 0.344	F = 1.30; *p* = 0.296	F < 1; *p* = 0.828

* Denotes significant effect of treatment; ^#^ denotes significant interaction; pg/mL: picograms/millimeters.

**Table 4:** Neurotrophic factors comparison (^Δ^ scores). Data are mean and SD and percentage (%).

	WB-EMS		no WB-EMS		Intervention	Time	Interaction
	Post15	Post60	Post15	Post60	F (1,9)	F (1,9)	F (1,9)
^Δ^BDNF (pg/mL)	4.1 (220.8)	110.3 (207.3)	130.4 (223.2)	102.1 (209.9)	F < 1; *p* = 0.466	F < 1; *p* = 0.438	F = 1.44; *p* = 0.260
^Δ^FGF21 (pg/mL)	−51.6 (126.2)	−27.5 (261.1)	274.5 (538.4)	79.2 (354.9)	F = 3.1; *p* = 0.111	F = 3.3; *p* = 0.100	F = 3.2; *p* = 0.107
^Δ^proNGF (pg/mL) *^, #^	−574.6 (569.1)	−256.9 (721.1)	390.2 (559.5)	−30.0 (385.0)	F = 6.8; *p* = 0.027	F < 1; *p* = 0.631	F = 12.2; *p* = 0.007
^Δ^mNGF (pg/mL)	−35.1 (59.8)	−44.5 (102.8)	−61.5 (218.2)	−46.1 (173.1)	F < 1; *p* = 0.786	F < 1; *p* = 0.835	F < 1; *p* = 0.504
^%^BDNF (%)	2.7 (12.5)	2.1 (16.9)	7.9 (10.8)	5.4 (18.1)	F < 1; *p* = 0462	F < 1; *p* = 0.704	F < 1; *p* = 0.759
^%^FGF21 (%)	−6.7 (21.6)	6.0 (43.5)	47.2 (94.4)	20.2 (54.1)	F = 3.4; *p* = 0.096	F < 1; *p* = 0.389	F = 3.4; *p* = 0.098
^%^proNGF (%)	−39.0 (33.3)	16.0 (84.1)	225.2 (532.6)	−16.2 (214.4)	F = 1.1; *p* = 0.312	F = 3.4; *p* = 0.096	F = 4.1; *p* = 0.074
^%^mNGF (%)	−13.5 (32.6)	−23.2 (52.8)	35.1 (103.4)	21.0 (165.8)	F = 1.2; *p* = 0.291	F < 1; *p* = 0.623	F < 1; *p* = 0.905

* Denotes significant effect of treatment; ^#^ denotes significant interaction; pg/mL: picograms/millimeters.

## Data Availability

The data presented in this study are available on request from the corresponding author. The data are not publicly available due to sensitive information.
